# A Digital Approach to Denture Marking

**DOI:** 10.7759/cureus.98130

**Published:** 2025-11-30

**Authors:** Modhupa Ghosh, Rekha Gupta, Preeti N Mishra, Aditi Mishra, Smiti Bhardwaj, Shubhra Gill, Mamta Phogat

**Affiliations:** 1 Prosthodontics, Department of Dentistry, Guru Teg Bahadur Hospital, Delhi, IND; 2 Prosthodontics, Maulana Azad Institute of Dental Sciences, Delhi, IND; 3 Dental Implant Development Project, Maulana Azad Institute of Dental Sciences, Delhi, IND; 4 Orthodontics, Department of Dentistry, Guru Teg Bahadur Hospital, Delhi, IND

**Keywords:** denture marker, digital, forensic, intra-oral, near-field communication (nfc) tags, wireless

## Abstract

Critical situations demand an easy and swift identification of those affected. Dentures present in the oral cavity survive in catastrophic situations that the extremities are unable to. Use of denture markers is a very utilitarian means of identifying dentures as well as their users. With the advent of the digital era, the conventional modes of denture labelling, such as engraving, stainless-steel bands, photographs, and lenticular cards, fall short of the mark and no longer suffice. Near field communication is an upcoming wireless, digital, contactless, and economical alternative with lots of potential. It is an offset of the well-known Radio Frequency Identification technology, but with fewer hassles. This case report presents the usage of NFC tags as denture markers within patients’ dentures and a six-month follow-up of their performance intraorally.

## Introduction

An individual’s identity is his fundamental right, which is of absolute importance both during his lifetime as well as post demise. It is the society’s moral obligation to provide dignity beyond life with the basic tenet of an identity. Forensic science, for a long time, has been trying to provide various means to fulfill the same. Forensic odontology has become an integral part of forensic science. This branch of forensics engages in processing and evaluation of the dental evidence with the purpose of delivering justice [1]. The occurrence of mass disasters, both natural and man-made, such as earthquakes, hurricanes, floods, acts of terrorism, and air crashes, is numerous, wide-ranging, and well-documented. Following such incidents, a definitive and early identification of the dead and injured becomes of utmost importance. The oral cavity’s ability to resist high temperatures helps preserve many key features that facilitate identification [2]. In the absence of a comprehensive fingerprint and DNA database, in many countries, forensic dentistry may represent the only way to obtain a positive identification.

For those having part of their dentition present, the restorations, missing teeth, or any fixed prosthesis present may act as a source of identification. However, the same is not applicable to those devoid of any dentition. Cheiloscopy and rugoscopy are alternatives that can be used, but only to a limited extent [3]. Use of labeled dentures, both partial and complete, is a viable option in such cases. Denture marking serves the dual purpose of identifying individuals as well as dentures by providing vital clues. The conventional methods of denture labeling encompass a variety of surface marking and inclusion methods such as engraving, scribing, use of photographs, stainless steel bands, bar codes, and lenticular cards [4,5]. More recently, radio frequency identification (RFID) tags have been advocated as a promising alternative. However, the price of the tags, along with the need for an expensive RFID reader, preclude its use as a cost-effective option [6,7].

This case report describes the use of a near field communication (NFC) tag as a potential alternative for the purpose of marking dentures. With the rapid ingress of digitization in almost all walks of life, especially healthcare, an effort was made to incorporate this contemporary, cost-effective, wireless technology as a denture marker and evaluate its practical efficiency in vivo. The objective was to assess the durability and functional capacity of an NFC tag, embedded within a denture, under clinical conditions, thereby determining its potential as a reliable identification method.

## Case presentation

Patients with at least one removable complete denture, possessing sufficient cognitive capacity to follow instructions, and having the manual dexterity to perform denture hygiene protocols were the target population. The tags were placed in two patients who reported to the Department of Prosthodontics for the fabrication of complete dentures. They were educated about the process of denture labeling using NFC tags, and informed consent was taken. The conventional series of steps of primary impression, final impression, recording of maxillomandibular relation, wax trial, curing, followed by finishing was performed. The final visit was scheduled when the dentures were incorporated with the programmed NFC tag.

**Figure 1:**
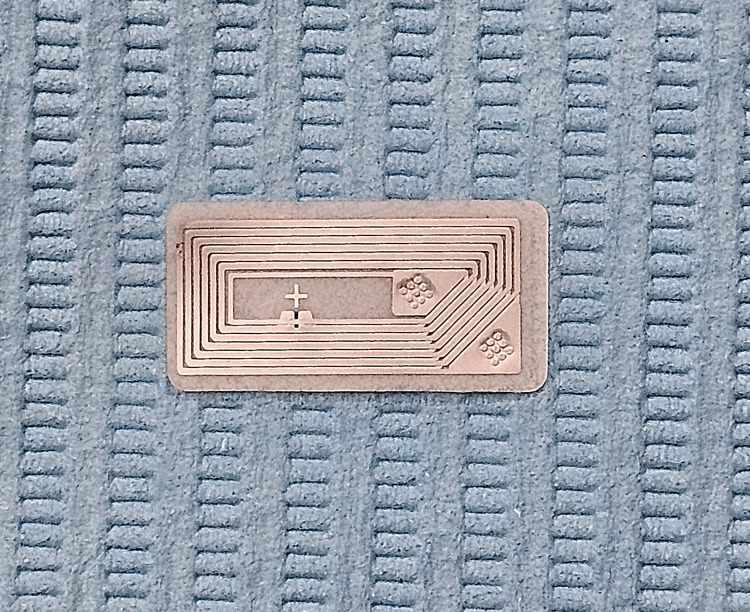
Near field communication (NFC) tag.

**Figure 2:**
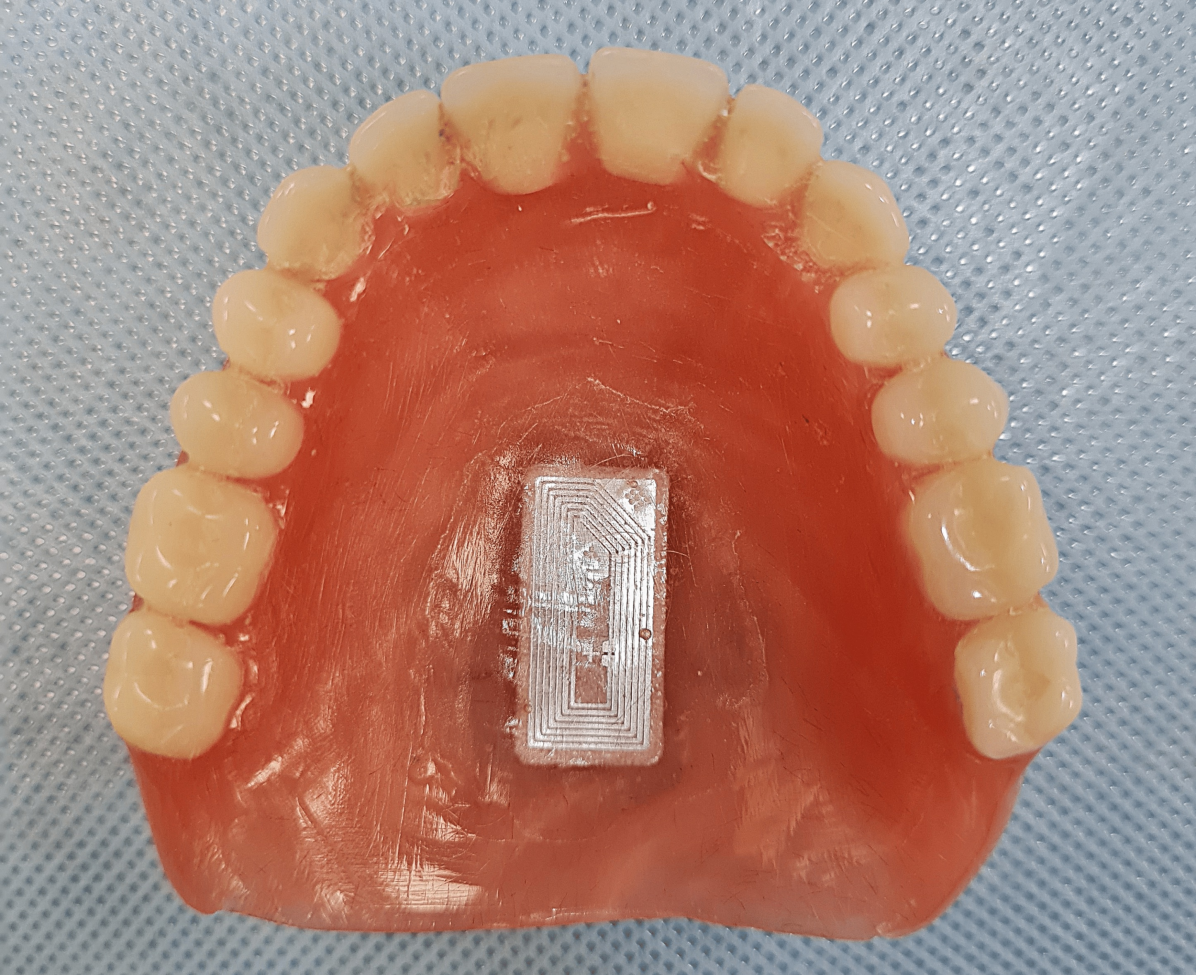
NFC tag incorporated in the palatal region of the maxillary denture.

**Figure 3:**
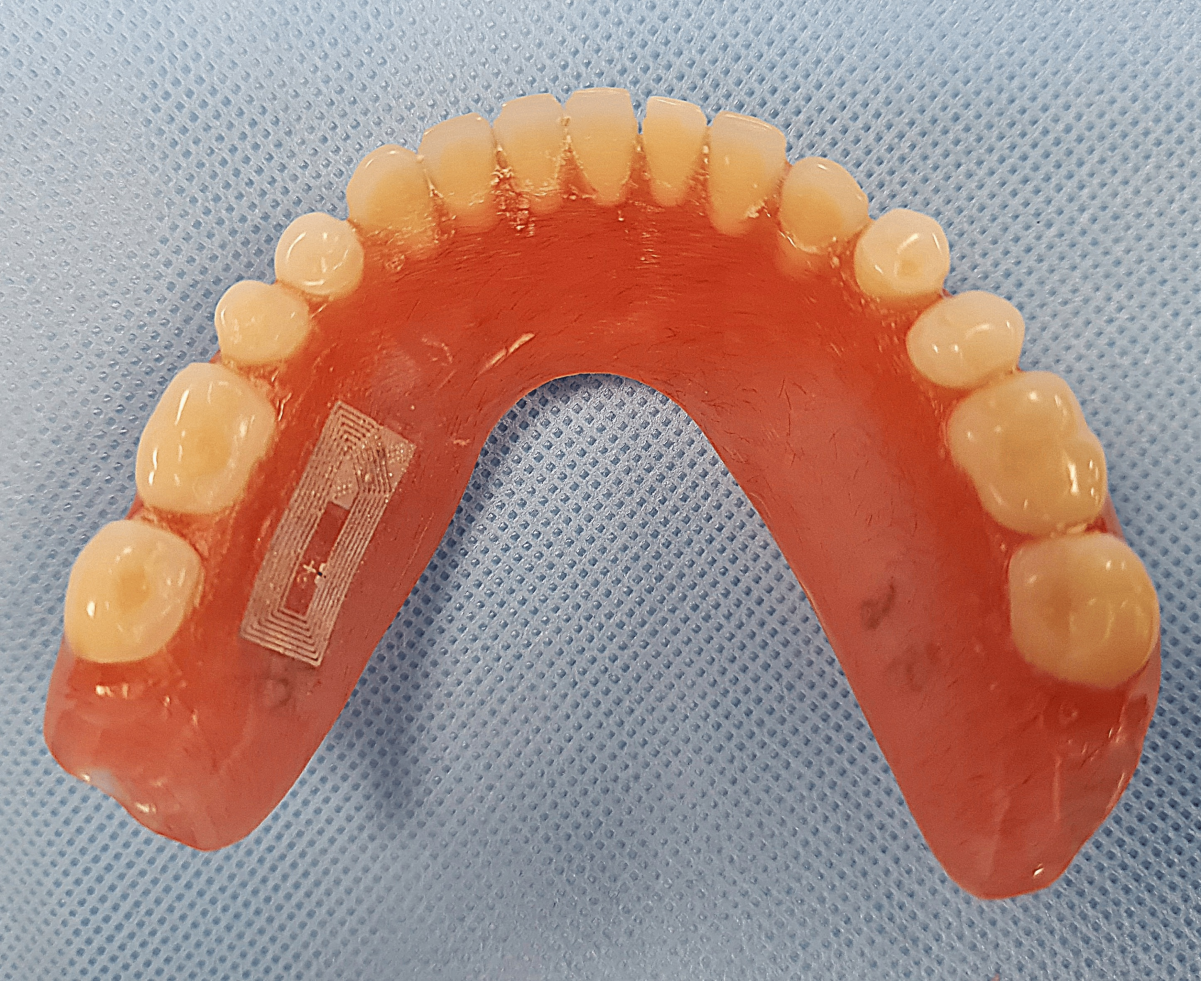
An NFC tag is incorporated in the lingual flange of the mandibular denture.

An NFC-enabled smartphone (Samsung Galaxy Note 20; Samsung) was utilized. The appropriate application (NFC tools; Wakdev) was downloaded from the Play Store (Google Play; Google Inc.) available on the smartphone. The application was used to program the NFC tags (NTAG216; LINQS) (Figure 1) with patient details such as name, age, sex, contact number, universal ID number, and hospital registration number. An 8 mm × 18 mm trough, following the outline of the tag, was marked and then created on the palatal aspect of the maxillary denture (Figure 2) of the first case and the lingual flange of the mandibular denture (Figure 3) of the second case. Subsequently, the tags were placed within the trough and sealed using clear self-polymerizing resin. The dentures were then finished to a fine polish.

**Figure 4:**
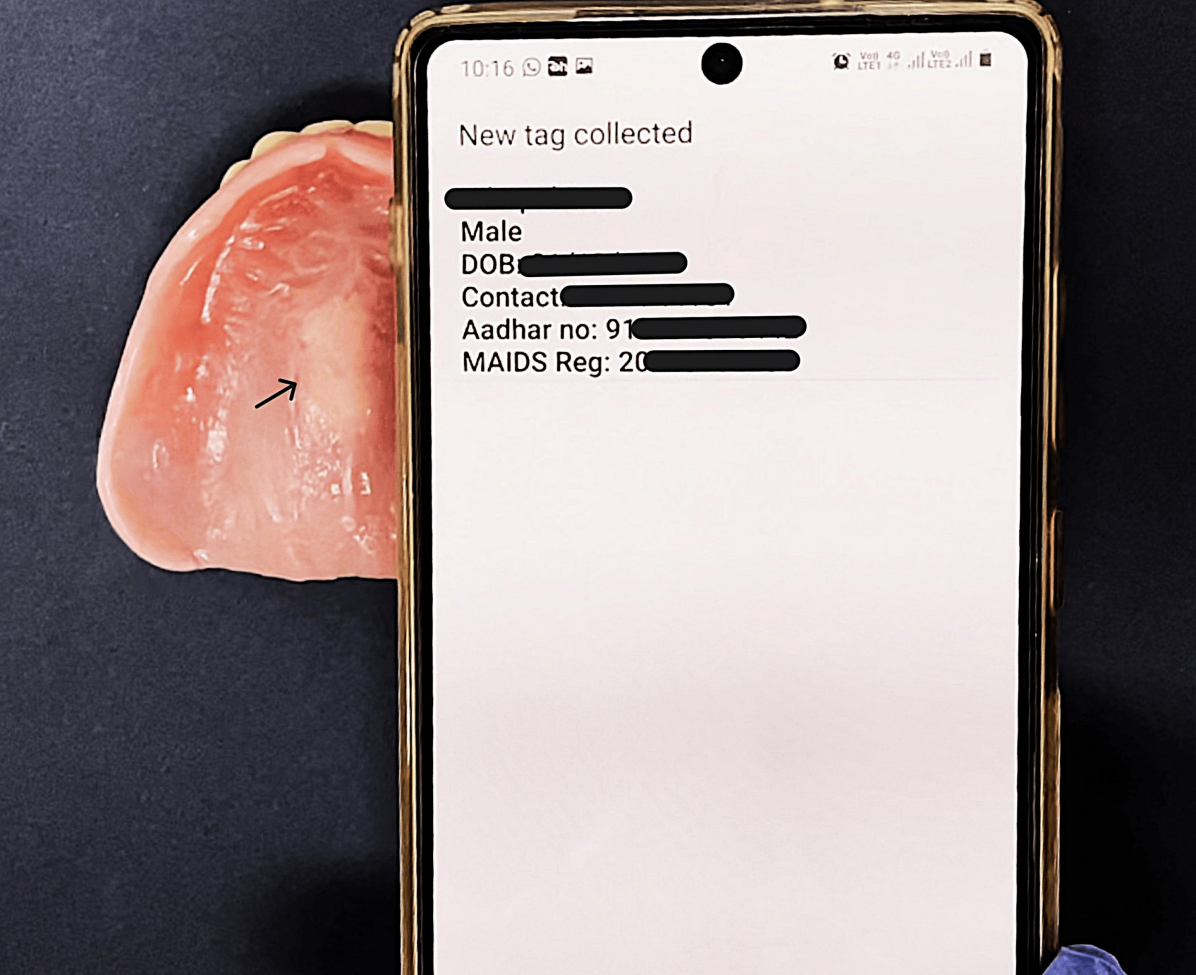
Patient details incorporated in the NFC tag successfully displayed on the smartphone screen. Arrow indicates the NFC tag embedded within the denture.

Post insertion, the working of the tags was appraised by bringing the dentures close to the NFC-enabled smartphone and scanning the tags. A successful reading of the tags resulted in the incorporated details being displayed on the smartphone’s screen (Figure 4).

The finished and polished dentures were then seated in the patient’s mouth. 

The patients were instructed to use the dentures in the usual manner, follow their routine food practices, regularly clean the dentures, and store them in water when not in use. The intended purpose was to subject the tags to a variety of thermal and chemical insults in terms of food intake at varying temperatures and regular disinfection protocols being followed. The patients were kept on follow-up. Subsequently, the tags were checked for survival again after one month and six months and were found to be functional despite their intraoral location and normal denture usage by the patients (Table 1). No tag failure was observed.

**Table 1:** Survival of the NFC tags after one and six months. + : Positive data retrieval

NFC tag	Survival after 1 month	Survival after 6 months
Case 1 (Maxillary denture)	+	+
Case 2 (Mandibular denture)	+	+

## Discussion

Critical situations call for an easy and early identification of the victim. In case of an emergency, timely access to pertinent details can be the difference between life and death. Dentures present in the oral cavity usually survive catastrophic situations, which the extremities are unable to, as they are shielded by the surrounding oral musculature, bones, and teeth [2]. Labelling of dentures can be of immense use in such scenarios. Nowadays, it is an acknowledged means of identifying individuals and misplaced dentures in countries such as Sweden, Scandinavia, Australia, and many states of America [8]. Geriatric institutions, nursing homes, dental laboratories, unconscious individuals, and patients suffering from mental illness or loss of memory can benefit immensely from this practice. Moreover, with the increase in globalization, people shifting base to different cities or continents has become a common phenomenon. The issues associated with the relocation of individuals globally can also be addressed, as the data stored in a labelled denture may also help in conveying pertinent information to the new treating doctor [9-13]. At present, none of the known methods can fulfill all the criteria of a suitable marker.

Near field communication (NFC) is a recent, wireless, digital subsection of the FDA-approved high-frequency RFID, operating at a frequency of 13.56 Hz. It is a battery-less, non-contact technology that allows devices to connect when in a proximity of approximately 10 cm. These compact-sized tags act as a memory chip [7,14]. They are passive in nature and become active when in close contact with an NFC-enabled smartphone by magnetic induction coupling. With smartphones becoming omnipresent and most of them being NFC enabled, the data can be accessed, added, and edited wirelessly and repeatedly through freely available online applications, eliminating the need for an additional reader [15]. The absence of a separate reader makes this approach significantly more practical and economical compared with traditional RFID tag systems. Additionally, access to the data stored in NFC is not reliant on internet availability or on the strength of network signals.

These tags have a substantial internal storage capacity and have features to lock data permanently in place. They are also equipped with password-based protections to prevent unauthorized access. They can be easily read even when embedded within the denture and need not be visible, unlike QR codes, which must be exposed for scanning. The small size, thinness, and flexibility of the tags make them very easy to adapt and incorporate within the denture. No interference with acrylic resin polymerization was observed with the post-fabrication inclusion technique used in both cases. The very economical price of the NFC tags also makes them a cost-effective option. These tags have been found to be resistant to various acids, alkalis, and thermal insults, which again showcases their durability [16]. The tags in this case report were exposed to a variety of harsh conditions intraorally, which were unconducive to their survival, but still remained functional after one and six months, thus proving their efficiency despite their delicate appearance. However, the long-term durability and functionality of these tags in the oral environment, particularly in saliva, remain unknown, and studies with extended follow-up are needed to evaluate their performance over time.

## Conclusions

The purpose of this case report was to provide an insight into the untapped potential of NFC tags. Though tested in a limited population, the successful survival of these tags within the challenging environment of the oral cavity suggests that they may be a promising alternative to the conventional denture markers. At present, this concept is still in its infancy, and further long-term studies with a larger sample size need to be carried out to validate its efficacy.
